# Clinical efficacy, safety and tolerability of Aliskiren Monotherapy (AM): an umbrella review of systematic reviews

**DOI:** 10.1186/s12872-020-01442-z

**Published:** 2020-04-17

**Authors:** Qiyuan Zhao, Jiantong Shen, Jingya Lu, Qi Jiang, Yuanyuan Wang

**Affiliations:** 1 School of Nursing, Huzhou University, Huzhou Central Hospital, 759 Erhuan Rd, Huzhou, 313000 Zhejiang People’s Republic of China; 2 School of Medicine, Huzhou University, Huzhou Central Hospital, 759 Erhuan Rd, Huzhou, 313000 Zhejiang People’s Republic of China

**Keywords:** Aliskiren, Monotherapy, Clinical effectiveness, Safety

## Abstract

**Background:**

Aliskiren is a newly developed drug. Its role in lowering BP has been recognized. However, the role of aliskiren in treating heart and renal diseases are still controversial.

**Objective:**

To evaluate the existing evidence about clinical efficacy, safety and tolerability of aliskiren monotherapy (AM).

**Methods:**

An umbrella review of systematic reviews of interventional studies. We searched Pubmed, Embase and Cochrane Library up to June 2019. Two reviewers applied inclusion criteria to the select potential articles independently. The extract and analyze of accessible data were did by two reviewers independently too. Discrepancies were resolved with discussion or the arbitration of the third author.

**Results:**

Eventually, our review identified 14 eligible studies. Results showed that for essential hypertension patients, aliskiren showed a great superiority over placebo in BP reduction, BP response rate and BP control rate. Aliskiren and placebo, ARBs or ACEIs showed no difference in the number or extent of adverse events. For heart failure patients, AM did not reduce BNP levels (SMD -0.08, − 0.31 to 0.15) or mortality rate (RR 0.76, 0.32 to 1.80), but it decreased NT-proBNP (SMD -0.12, − 0.21 to − 0.03) and PRA levels (SMD 0.52, 0.30 to 0.75), increased PRC levels (SMD -0.66, − 0.8 to − 0.44). For patients who are suffered from hypertension and diabetes and/or nephropathy or albuminuria at the same time, aliskiren produced no significant effects (RR 0.97, 0.81 to 1.16).

**Conclusion:**

We found solid evidence to support the benefits of aliskiren in the treatment of essential hypertension, aliskiren can produce significant effects in lowering BP and reliable safety. However, the effects of aliskiren in cardiovascular and renal outcomes were insignificant.

**Trial registration:**

Study has been registered in PROSPERO (CRD42019142141).

## Background

Hypertension, as a highly prevalent disease and its control is still unsatisfactory. The prevalence of hypertension in the United States (defined as taking antihypertensive medication or having a systolic pressure of ≥140 mmHg and/or a diastolic pressure ≥ 90 mmHg) was approximately 32% and had remained relatively constant since 1999 [1–3]. HF (heart failure) is also a rapidly growing public health problem, the estimated prevalence of it is > 37.7 million individuals globally, it has creating a great burden to society [4].

By targeting blood pressure (BP) and related abnormalities of the heart and blood vessels, renin-angiotensin-aldosterone system (RAAS) inhibitors can prevent target organ damage and related cardiovascular events [5]. Blockade of renin has already been recognized as the optimal means for inhibiting the RAAS [6]. Aliskiren is the first one in a new class of oral, non-peptide, low molecular weight direct renin inhibitors (DRI) [7]. It has already been approved by the US Food and Drug Administration (FDA) and some European countries for the treatment of essential hypertension [8]. As reported, aliskiren produced good results on certain surrogate end-points in HF setting: BNP (brain natriuretic peptide) levels, N-terminal prohormone of BNP (NT-proBNP) levels, plasma renin activity (PRA), plasma renin concentration (PRC), etc [9] Aliskiren is also reported to have good effects on the renal function, as it can increase renal blood flow, and may prevent the deterioration of renal [10, 11].

Systematic reviews, meta-analyses and pooled analyses of interventional studies have evaluated the efficacy, safety and tolerability of aliskiren. However, in contrast with the promising prospect, several reviews showed that aliskiren presented no significant influence on several important clinical outcomes. In the present work, we carried out an umbrella review of the evidence across existing systematic reviews, meta-analyses and pooled analyses of interventional studies that reported clinical outcomes after using aliskiren monotherapy (AM).

Our overview was aimed to provide an overview of the range and validity of the reported associations between AM and clinical efficacy as well as the side effects. We compared aliskiren with placebo and other pharmaceutical drugs: Angiotensin Receptor Blockers (ARBs), Angiotensin-Converting Enzyme Inhibitors (ACEIs), hydrochlorothiazide (HCT/HCTZ), etc. to evaluate the effects of cardiovascular outcomes and renal effects between AM and other antihypertensive (BP reduction, BP response rate, BP control rate, the incidence of some adverse events).

## Methods

### Search strategy and selection criteria

We searched Pubmed, Embase and Cochrane Library, from inception until June 2019 to identify systematic reviews, meta-analyses and pooled analyses of interventional studies investigating associations between AM and clinical outcomes. The search strategy as follows: (aliskiren OR direct renin inhibitor OR renin-angiotensin inhibition OR spp100 OR takturna) AND (systematic review OR meta-analysis OR pooled analysis). We also hand-searched all reference lists of the included studies to identify additional reviews of relevance. We used Endnote X9 to screen literatures. Two researchers screened the titles and abstracts independently, then reviewed the full text of selected articles and evaluated their eligibility. Any discrepancies were resolved with discussion or the arbitration of the third author.

### Inclusion and exclusion criteria

We included English articles; The meta-analysis, systematic review or pooled analysis; meta-analyses that integrated the randomized controlled trials (RCT) which evaluated efficacy, tolerability or safety of AM. The latest edition was selected priority. Articles that had substantial data were included.

Observational studies, conference briefs, editorials and overview articles were excluded. We also excluded meta-analyses in which the intervening measures only included aliskiren combination therapy, or the primary outcome was not related to clinical efficacy, safety or tolerability. Meta-analyses that did not provide specific study data [number of incident events, number of study population, follow-up period, relative risks and 95% confidence intervals (CI)] and in which the missing data was not retrievable from the original studies were also excluded.

### Data extraction

Data extraction tables were established in Microsoft software. Two researchers extracted the data independently and discrepancies were resolved with consensus, if necessary, a third author would be involved. For each eligible article, extraction information as follows: the first author, year of publication, number of trials included, comparative drugs, follow-up period and the investigated clinical outcomes. We also calculated the study specific risk estimates [i.e., risk ratio (RR), odds ratio (OR), weighted mean difference (WMD), standard mean difference (SMD)] together with their 95% CI and number of incident events and total events in each study.

If more than one meta-analysis compared aliskiren with the same drug evaluating the same outcome, we will incorporate all the original trials from those meta analyses, without including duplicates. All the available data were synthesized to get more comprehensive and objective result.

### Data analysis

For each outcome, If the random model was not used, we will extracted data and estimated 95% CI using random effects methods by ourselves. Then calculated 95% predication intervals (PI) for each random effect estimate, to represent the range in which the effect estimates of future studies will lie [12].

If possible, we would stratify the comparisons into several groups according to the dose, such as low group, low to high group, high group. In each group, the doses of aliskiren and control drug are comparable. We also stratified the comparisons according to the categories of contrast drugs. For example, we divided ARBs into three specific drugs: losartan, valsartan and irbesartan.

Between-study heterogeneity was quantified using the *I*^*2*^ metric. *I*^*2*^ ranges between 0 and 100% and quantifies the variability in effect estimates that is due to heterogeneity rather than sampling error [13]. Values exceeding 50% or 75% are considered to represent substantial or considerable heterogeneity.

Furthermore, if an estimate included at least 3 articles, we would reanalyse the estimate with Egger’s asymmetry test, to detect and visualize the possible publication bias in the article. Revman and STATA 14.0 were used.

### Evaluation of quality of included studies

We evaluated the quality of all included studies using the AMSTAR 2 tool, a comprehensive critical appraisal instrument that evaluated different aspects of reviews, to distinguish high quality ones [14].

### Patient and public involvement

No patients will be involved in developing plans for project and implementation of the study. None of them will be asked to advise on interpretation of results. The results will be disseminated to the ordinary population through public presentations by the authors.

## Results

### Eligible studies

**Fig. 1:**
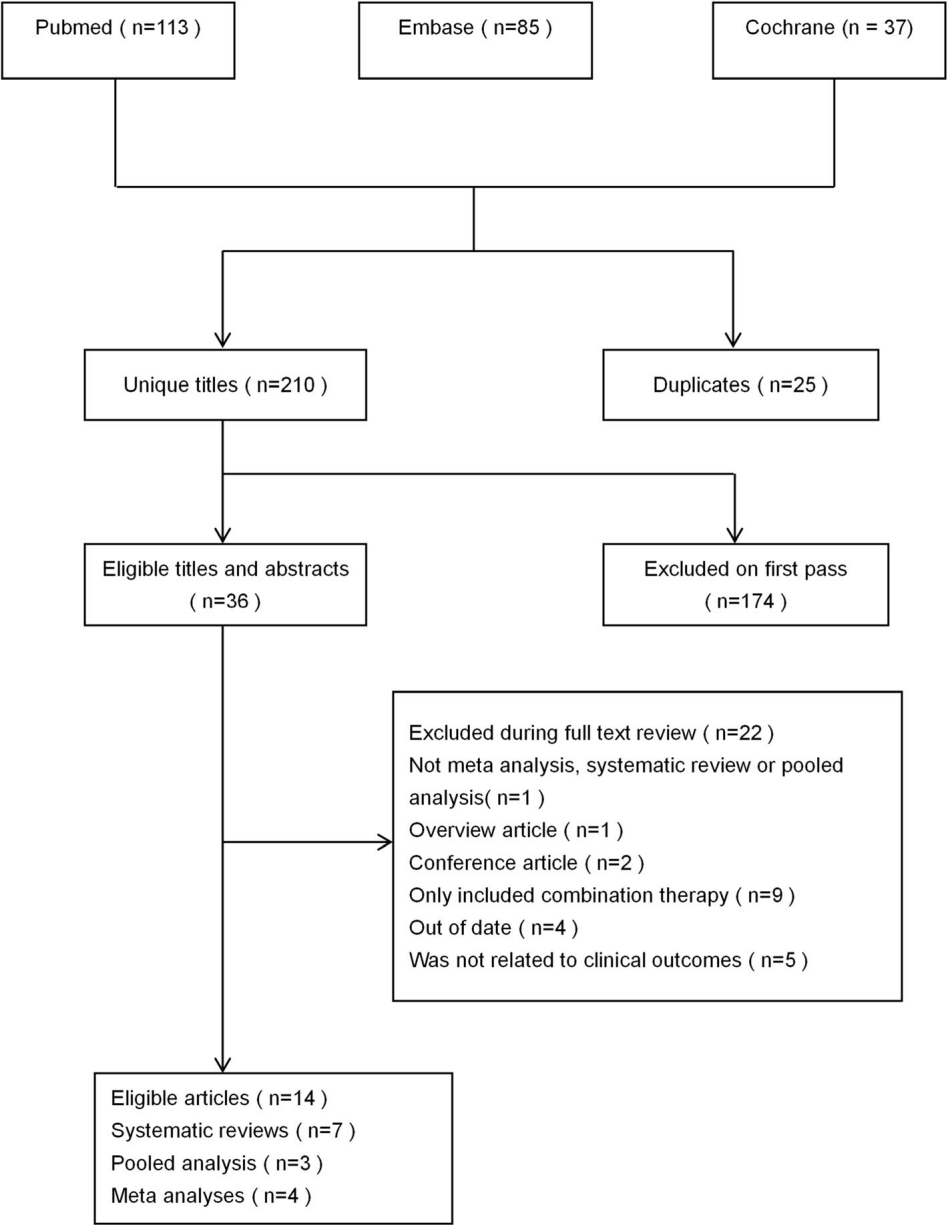
Flowchart of selection of studies for inclusion in umbrella review on AM and clinical outcomes

The literature search yielded 235 articles, of which 14 articles met our inclusion standard (see Fig. 1). Eleven are meta analyses or systematic reviews, only three are pooled analyses, all of them are the analyses of RCTs. The included articles were published from 2010 to 2019.

The 14 eligible articles contained a large number of meta-analyses and several unique outcomes. These meta-analyses are the comparisons between aliskiren and other pharmaceutical drugs, in aim of evaluating the association between AM and antihypertensive effects, the incidence of adverse events (for hypertension patients), cardiovascular outcomes (for HF patients) and renal effects (for different types of patients). More than one measurement index would be included for each outcome,.

### Antihypertensive effects

To evaluate clinical value of AM in essential hypertension patients, we compared aliskiren with other antihypertensive drugs in four ways, including BP reduction, BP response rate, BP control rate, the incidence of adverse events.

#### BP reduction

When comparing aliskiren to placebo, according to the dose of aliskiren used (75 mg, 150 mg, 300 mg, 600 mg), we stratified the comparisons into four groups [8, 15]. Independent of the dose, aliskiren reduced BP to a greater degree. After using aliskiren for 8–26 weeks, both diastolic blood pressure (DBP) and systolic blood pressure (SBP) dropped significantly.

When comparing aliskiren to ARBs, we divided the comparisons into three groups: low dose group (aliskiren 150 mg), low to high dose group (aliskiren 150-300 mg), high dose group (aliskiren 300 mg) [16, 17]. In all three groups, the doses of aliskiren and ARBs were comparable. However, all the results showed that reductions from baseline to endpoint in both DBP and SBP did not differ between these two drugs.

When comparing aliskiren to ACEIs in the effects of BP reduction, our study included two meta analyses [15, 18]. Aliskiren was slightly superior to ACEIs in reducing both DBP and SBP.

Aliskiren was inferior to amlodipine in reducing BP. Aliskiren and HCTZ showed no difference in BP reduction. Aliskiren was inferior to atenolol in reducing DBP, though two drugs showed no difference in SBP reduction.

When comparing aliskiren150mg to aliskiren75mg, aliskiren300mg to aliskiren150mg. With an increase of dosage, the effect of lowering DBP and SBP both significantly improved. However, according to the results, aliskiren 300 mg and 600 mg had similar effects in lowering BP [see Table 1 Reductions in mean sitting DBP (msDBP) and mean sitting DBP (msSBP)].

**Table 1:** Reductions in mean sitting DBP (msDBP) and mean sitting DBP (msSBP)

Outcome	No. of SRs	No. of original studies	No. of cases/ controls	Follow-up range (weeks)	Estimate (95%CI)	*P- value*	95%PI	*I*^*2*^(P*)	Egger’s *P* value
**msDBP**									
aliskiren75mg vs placebo	1	5	821/279	8–13	− 2.05 [− 3.13,-0.96]	< 0.001	[− 3.81,-0.28]	0%(0.73)	0.043
aliskiren150mg vs placebo	1	12	2665/1118	8–26	−3.19 [−4.02, -2.37]	< 0.001	[−5.57,-0.82]	47%(0.04)	0.798
aliskiren300mg vs placebo	1	10	2193/808	8–26	−4.51 [−5.27, −3.76]	< 0.001	[− 5.97, − 3.06]	17%(0.29)	0.359
aliskiren600mg vs placebo	1	2	296/97	8	−5.86 [−7.73, -3.99]	< 0.0001	NA	0%(0.57)	NA
aliskiren vs ARBs (low dose)	2	4	648/532	4–8	0.07 [−0.94,1.09]	0.89	[−2.15,2.30]	0%(0.48)	0.12
aliskiren vs ARBs (low to high dose)	2	5	923/944	8–12	−0.25 [−2.32,1.82]	0.81	[−7.82,7.31]	82% (0.0002)	0.272
aliskiren vs ARBs (high dose)	2	3	241/122	8	−0.65 [−2.52, 1.23]	0.5	[−12.79,11.49]	0%(0.89)	0.293
aliskiren vs ACEIs	2	3	796/786	≥8	−1.19[− 1.99, −0.38]	0.004	[−6.42,4.05]	0%(0.53)	0.687
aliskiren vs amlodipine	1	1	203/181	8	3.63 [1.85,5.41]	< 0.0001	NA	NA	NA
aliskiren vs HCTZ	1	1	183/176	8	−0.9 [−2.56,0.76]	0.29	NA	NA	NA
aliskiren vs atenolol	1	1	231/231	12	2.4 [0.74,4.06]	0.004	NA	NA	NA
Aliskiren150mg vs 75 mg	1	5	830/824	8–13	−0.8 [−1.58,-0.03]	0.04	[−2.06,0.04]	0%(0.45)	0.944
aliskiren300mg vs 150 mg	1	10	2193/2195	8–26	−1.75 [−2.31, − 1.20]	< 0.001	[− 2.88,-0.63]	20%(0.26)	0.584
aliskiren600mg vs 300 mg	1	2	296/296	8	−0.68[−2.03,0.67]	0.449	NA	33%(0.22)	NA
**msSBP**									
aliskiren75mg vs placebo	1	5	821/279	8–13	−2.97[−4.76, -1.18]	0.001	[−5.88,-0.66]	0%(0.57)	0.253
aliskiren150mg vs placebo	1	12	2665/1121	8–26	−5.93[−6.94, -4.91]	< 0.001	[−7.93,-3.92]	17%(0.28)	0.674
aliskiren300mg vs placebo	1	10	2199/810	8–26	−7.91 [−9.15, −6.67]	< 0.001	[−10.48,-5.33]	22%(0.24)	0.693
aliskiren600mg vs placebo	1	2	296/97	8	−11.35[− 14.43,-8.27]	< 0.00001	NA	0%(0.62)	NA
aliskiren vs ARBs (low dose)	2	4	648/532	4–8	1.25 [−0.29,2.78]	0.111	[−2.57,5.06]	6%(0.36)	0.661
aliskiren vs ARBs (low to high dose)	2	5	923/944	8–12	−1.19[−4.09, 1.70]	0.42	[−11.46,9.08]	77% (0.002)	0.242
aliskiren vs ARBs (high dose)	2	3	241/122	8	0.24 [−2.29, 2.76]	0.85	[−16.10,16.57]	0%(0.65)	0.841
aliskiren vs ACEIs	2	4	1253/1230	≥8	−2.37[−3.48, −1.26]	< 0.0001	[−4.83,0.04]	0%(0.59)	0.123
aliskiren vs amlodipine	1	1	203/181	8	5.67 [2.86,8.48]	< 0.0001	NA	NA	NA
aliskiren vs HCTZ	1	1	183/176	8	−1.4 [−4.04,1.24]	0.3	NA	NA	NA
aliskiren vs atenolol	1	1	231/231	12	−0.08 [−3.02,2.86]	0.96	NA	NA	NA
aliskiren150mg vs 75 mg	1	5	830/821	8–13	−1.89 [−3.34, −0.43]	0.011	[−5.37,1.59]	23%(0.27)	0.872
aliskiren300mg vs 150 mg	1	10	2199/2195	8–26	−2.57 [−3.72, −1.42]	< 0.0001	[− 5.87,0.73]	52%(0.03)	0.921
aliskiren600mg vs 300 mg	1	2	296/296	8	−0.61 [−2.78, 1.56]	0.581	NA	0%(0.60)	NA

**Notes:** Type of metric: WMD (Weighted mean difference)

Abbreviations: *NA* Not Accessible, *SR* Systematic review

#### BP response rate

BP response rate is defined as the percentage of patients with a mean DBP < 90 mmHg and/or at least 10 mmHg reduction from baseline [15]. When compared with placebo, aliskiren created statistically significant improvements on response rates in all doses. When compared with amlodipine and atenolol, aliskiren resulted in fewer patients receiving BP response. Aliskiren and ARBs, ACEIs, HCTZ showed comparable results in the proportion of patents received BP response (see Table 2 BP response rate and BP control rate).

**Table 2:** BP response rate and BP control rate

Outcome	No.of SRs	No.of original studies	No.of cases/ controls	Follow-up range (weeks)	Estimate (95%CI)	*P- value*	95%PI	*I*^*2*^(P*)	Egger’s *P* value
**BP response rate**									
aliskiren 75 mg vs placebo	2	2	292/291	8	1.41 [1.03–1.94]	0.03	NA	62%(0.11)	NA
aliskiren150mg vs placebo	2	3	460/451	8	1.42 [1.16–1.75]	0.001	[0.19–10.87]	43%(0.17)	0.008
aliskiren300mg vs placebo	2	7	1443/1480	8	1.65 [1.46–1.85]	< 0.001	[1.17–2.35]	49%(0.07)	0.167
aliskiren vs ARBs (low dose)	2	2	479/361	4–8	0.92 [0.81–1.04]	0.16	NA	0%(0.89)	NA
aliskiren vs ARBs (low to high dose)	2	2	543/570	8–12	0.99 [0.90–1.09]	0.88	NA	0%(0.51)	NA
aliskiren vs ARBs (high dose)	1	1	175/60	8	1.07 [0.86–1.33]	0.52	NA	NA	NA
aliskiren vs ACEIs	1	1	414/418	8–26	1.10 [0.99–1.24]	0.09	NA	NA	NA
aliskiren vs amlodipine	1	2	513/492	8–32	0.77[0.69–0.85]	< 0.00001	NA	0% (0.37)	NA
aliskiren vs HCTZ	1	1	180/173	8	1.08 [0.92–1.28]	0.34	NA	NA	NA
aliskiren vs atenolol	1	1	230/230	12	0.84[0.74–0.95]	0.007	NA	NA	NA
**BP control rate**									
aliskiren 75 mg vs placebo	2	1	177/176	8	1.30 [0.95–1.77]	0.1	NA	NA	NA
aliskiren150mg vs placebo	2	3	475/666	8	1.51 [1.06–2.16]	0.02	[0.03–76.62]	62%(0.07)	0.084
aliskiren300mg vs placebo	2	6	1276/1288	8	1.62 [1.10–2.38]	0.01	[0.41–6.36]	90% (< 0.00001)	0.741
aliskiren vs ARBs (low to high dose)	2	5	919/921	8–12	1.05 [0.89–1.23]	0.57	[0.68–1.62]	37%(0.18)	0.357
aliskiren vs ARBs (high dose)	1	1	175/60	8	1.01 [0.72–1.43]	0.93	NA	NA	NA
aliskiren vs ACEIs	1	1	414/418	8–26	1.12 [0.96–1.30]	0.15	NA	NA	NA
aliskiren vs amlodipine	1	1	201/179	8	0.72 [0.57–0.91]	0.006	NA	NA	NA
aliskiren vs HCTZ	1	1	180/173	8	1.24 [0.97–1.59]	0.09	NA	NA	NA
aliskiren vs atenolol	1	1	230/230	12	0.86 [0.68–1.08]	0.18	NA	NA	NA

**Notes:** Type of metric for comparisons: RR (Risk ratio)

Abbreviations: *NA* Not Accessible, *NR* Not Report, *SA* Systematic review

#### BP control rate

BP control rate is defined as the percentage of patients with a mean DBP < 90 mmHg and mean SBP < 140 mmHg [15]. When compared with placebo, aliskiren resulted in a greater BP control rate in different doses, aside from aliskiren 75 mg. When compared with amlodipine, aliskiren resulted in a smaller BP control rate. Aliskiren and ARBs, ACEIs, HCTZ, atenolol showed comparable results in the proportion of patents received BP control (see Table 2).

#### The incidence of adverse events

When compared with placebo, according to the dose of aliskiren used (75 mg, 150 mg, 300 mg, 600 mg), we stratified the comparisons into four groups [8]. The results showed that aliskiren600mg resulted in more adverse events than placebo. Aliskiren150mg and 600 mg led to fewer withdrawals due to adverse events than placebo. Except for the above special cases, the incidence of total adverse events, severe adverse events and withdrawal due to adverse events are comparable between aliskiren and placebo.

When compared with ARBs, aliskiren showed no difference between two drugs in the number or extent of adverse events [16]. Then we divided ARBs into three specific drugs: losartan, valsartan and irbesartan, the results were unchanged.

When compared aliskiren with some active comparators [15, 19] (ACEIs, amlodipine, HCTZ or atenolol), the results showed no difference in adverse events or in withdrawals due to adverse events (see Table 3 The incidence of adverse events, serious adverse events and withdrawal due to adverse events).

**Table 3:** The incidence of adverse events, serious adverse events and withdrawal due to adverse events

Outcome	No.of SRs	No.of original studies	No.of cases/controls	Follow-up range (weeks)	Estimate (95%CI)	*P-value*	95%PI	*I*^*2*^(P*)	Egger’s *P* value
**Adverse events**									
aliskiren 75 mg vs placebo	1	5	742/740	8–13	0.96 [0.84–1.08]	0.48	[0.78–1.17]	0% (0.56)	0.853
aliskiren150mg vs placebo	1	10	1785/1639	8–26	0.99 [0.90–1.08]	0.77	[0.89–1.10]	0% (0.65)	0.43
aliskiren300mg vs placebo	1	9	1461/1478	8–26	1.02[0.93–1.12]	0.68	[0.91–1.15]	0% (0.47)	0.876
aliskiren600mg vs placebo	1	3	499/494	8	1.24 [1.07–1.43]	0.004	[0.49–3.13]	0% (0.88)	0.083
aliskiren vs ARBs	1	NR	NR	4–8	0.98 [0.89–1.08]	0.68	NR	NR	NR
aliskiren vs lorsartan	1	NR	NR	4–8	1.03 [0.79–1.35]	0.83	NR	NR	NR
aliskiren vs valsartan	1	NR	NR	8	0.92[0.81–1.05]	0.2	NR	NR	NR
aliskiren vs irbesartan	1	NR	NR	7–8	1[0.81–1.23]	0.99	NR	NR	NR
aliskiren vs ACEIs	1	2	514/508	8–26	1[0.89–1.11]	0.93	NA	0%	NA
aliskiren vs Amlodipine	1	2	513/492	8–32	0.99 [0.81–1.11]	0.92	NA	37%	NA
aliskiren vs HCTZ	1	1	183/176	8	0.95 [0.74–1.22]	0.68	NA	NA	NA
aliskiren vs atenolol	1	1	231/231	12	0.88 [0.72–1.08]	0.23	NA	NA	NA
**Serious adverse events**									
aliskiren vs placebo	1	8	3633/1683	8–26	0.75 [0.27–2.05]	0.57	[0.07–7.71]	32%(0.17)	0.811
aliskiren vs ARBS	1	NR	NR	4–8	0.72 [0.36–1.46]	0.36	NR	NR	NR
aliskiren vs lorsartan	1	NR	NR	4–8	0.33 [0.01–8.18]	0.5	NR	NR	NR
aliskiren vs valsartan	1	NR	NR	8	0.63 [0.09–4.43]	0.65	NR	NR	NR
aliskiren vs irbesartan	1	NR	NR	7–8	0.55[0.18–1.67]	0.29	NR	NR	NR
**Withdrawal due to adverse events**									
aliskiren 75 mg vs placebo	1	5	821/817	8–13	0.69 [0.37–1.29]	0.24	[0.25–1.90]	0%(0.52)	0.008
aliskiren150mg vs placebo	1	7	1161/1154	8–26	0.37[0.16–0.84]	0.02	[0.05–2.75]	38%(0.14)	0.065
aliskiren300mg vs placebo	1	6	984/1001	8–26	0.60 [0.34–1.06]	0.08	[0.21–1.74]	12%(0.34)	0.365
aliskiren600mg vs placebo	1	1	166/165	8	0.14 [0.03–0.62]	0.009	NA	NA	NA
aliskiren vs ARBs	1	NR	NR	4–8	0.82 [0.54–1.25]	0.35	NR	NR	NR
aliskiren vs lorsartan	1	NR	NR	4–8	0.76[0.28–2.08]	0.6	NR	NR	NR
aliskiren vs valsartan	1	NR	NR	8	0.89 [0.50–1.58]	0.69	NR	NR	NR
aliskiren vs irbesartan	1	NR	NR	7–8	0.73[0.33–1.61]	0.43	NR	NR	NR
aliskiren vs ACEIs	2	3	1145/952	8–36	0.76 [0.45–1.28]	0.3	[0.00–149.77]	50%(0.13)	0.953
aliskiren vs Amlodipine	1	2	513/492	8–32	0.42 [0.08–2.30]	0.32	NA	65%	NA
aliskiren vs HCTZ	1	1	183/176	8	1.92 [0.59–6.27]	0.28	NA	NA	NA
aliskiren vs atenolol	1	1	231/231	12	0.6 [0.22–1.62]	0.31	NA	NA	NA

**Notes**: Type of metric for comparisons: RR (Risk ratio)

Abbreviations: *NA* Not Accessible, *NR* Not Report, *SA* Systematic review

In order to evaluate the paradoxical pressure rises after using aliskiren, we included a systematic review involving 4877 patients [20]. The results showed that when compared with placebo, increases in BP were less frequent in the aliskiren group. When compared with some active comparators (ARBs, ramipril or HCT), the incidence of BP increases with aliskiren was comparable (see Table 4 The incidence of paradoxical BP rises).

**Table 4:** The incidence of paradoxical BP rises

Outcome	No. of SRs	No. of original studies	No. of cases/controls	Follow-up range (weeks)	Estimate (95%CI)	*P*-value
Paradoxical blood pressure rise: ΔmsDBP> 5 mmHg						
aliskiren vs placebo	1	4	2110/902	8	0.27 [0.20–0.37]	< 0.00001
aliskiren vs ARBs	1	4	2110/701	4–9	0.86 [0.55–1.36]	0.53
aliskiren vs ramipril	1	2	2110/617	9–26	1.19 [0.69–2.04]	0.53
aliskiren vs HCT	1	1	2110/547	52	1.20 [0.68–2.13]	0.52
ΔmsDBP> 10 mmHg						
aliskiren vs placebo	1	4	2110/902	8	0.27 [0.15–0.48]	< 0.00001
aliskiren vs ARBs	1	4	2110/701	4–9	0.57 [0.27–1.20]	0.14
aliskiren vs ramipril	1	2	2110/617	9–26	0.93 [0.37–2.31]	0.87
aliskiren vs HCT	1	1	2110/547	52	0.70 [0.30–1.67]	0.42
ΔmsSBP> 10 mmHg						
aliskiren vs placebo	1	4	2110/902	8	0.31 [0.24–0.41]	< 0.00001
aliskiren vs ARBs	1	4	2110/701	4–9	0.97 [0.64–1.48]	0.9
aliskiren vs ramipril	1	2	2110/617	9–26	0.69 [0.47–1.01]	0.05
aliskiren vs HCT	1	1	2110/547	52	0.89 [0.57–1.38]	0.59
ΔmsSBP> 20 mmHg						
aliskiren vs placebo	1	4	2110/902	8	0.40 [0.24–0.67]	0.0005
aliskiren vs ARBs	1	4	2110/701	4–9	1.50 [0.62–3.61]	0.37
aliskiren vs ramipril	1	2	2110/617	9–26	0.66 [0.34–1.29]	0.22
aliskiren vs HCT	1	1	2110/547	52	0.87 [0.40–1.92]	0.74

**Notes:** Type of metric for comparsions: RR (Risk ratio)

Abbreviations: *SA* Systematic review

### Cardiovascular outcomes

In order to evaluate the efficacy of aliskiren in HF patients, our study included a most recent article written by Luo Y and Chen Q [21]. The study compared aliskiren with placebo in the changes in some biological markers, aliskiren and placebo did not differ in BNP levels or mortality. However, aliskiren led to a significantly decreased NT-proBNP level (SMD -0.12, − 0.21 to − 0.03). Furthermore, aliskiren led to a significant reduced PRA (SMD -0.66, − 0.89 to − 0.44) and increased PRC (SMD 0.52, 0.30 to 0.75). No significant difference between the two drugs in aldosterone levels either.

In a separate article, the authors compared aliskiren with placebo in incidence of related cardiovascular events like myocardial infarction and stroke [22]. Results showed no difference between these two drugs. However, the study included only one article and 613 patients, results need to be further discussed. Both articles evaluated the association betweeen AM and all-cause mortality. The results showed that aliskiren did not affect the frequency of death in HF patients.

In another included article [23], the authors evaluated combined cardiovascular disease (CVD) mortality and HF hospitalization, aliskiren and enalapril showed no difference in the outcomes (see Table 5 The efficacy of aliskiren in heart failure).

**Table 5:** The efficacy of aliskiren in heart failure

Outcome	No. of SRs	No. of original studies	No. of cases/controls	Follow-up range (weeks)	Type of metric	Estimate (95%CI)	*P*-value	95%PI	I^2^(P*)	Egger’s *P* value
aliskiren vs placebo										
NT-proBNP levels	1	3	975/956	12–48	SMD	−0.12 [−0.21,-0.03]	0.01	[−0.70,0.46]	0% (0.57)	0.814
BNP levels	1	2	151/143	6–12	SMD	−0.08 [−0.31,0.15]	0.49	NA	0%(0.76)	NA
Plasma renin activity	1	3	176/157	12–48	SMD	−0.66 [−0.89,-0.44]	< 0.0001	[−2.11,0.77]	0%(0.85)	0.648
Plasma renin concentration	1	2	167/149	6–26	SMD	0.52 [0.30,0.75]	< 0.0001	NA	0%(0.72)	NA
Aldosterone level	1	2	151/143	6–12	SMD	−0.09 [−0.32,0.14]	0.44	NA	0%(0.55)	NA
Mortality	2	3	1255/1250	≥12	RR	0.76 [0.32–1.80]	0.53	[0.00,3273.53]	24%(0.27)	0.498
Myocardial infarction	1	1	305/308	104	RR	0.13[0.02–1.00]	0.05	NA	NA	NA
Stroke	1	1	305/308	104	RR	0.25 [0.03–2.25]	0.22	NA	NA	NA
aliskiren vs enalapril										
Combined cardiovascular mortality and hospitalisation	1	1	2340/2336	36	RR	0.98 [0.90–1.06]	0.57	NA	NA	NA

### Renal effects

To evaluate renoprotective effects of aliskiren, our study included several articles. For essential hypertension patients, our study included two studies compared aliskiren with placebo, ARBs and HCT in changes of some closely related outcome indicators [20, 24]. All the results failed to reach statistical significance, aliskiren showed no difference three drugs. Hyperkalemia is also an important indication for evaluating renal effects. The occurrence of hyperkalemia is comparable between aliskiren and placebo, ARBs or HCT.

In different type of patients, our study included a meta analysis [25] comparing aliskiren to placebo in increases in serum creatinine (sCr) or decreases in estimated glomerular filtration rate (eGFR). The results showed aliskiren had renoprotective influence and led to fewer renal impairment events, though this did not reach statistical significance (RR 0.97,0.81 to 1.16) [21] (see Table 6).

**Table 6:** Aliskren monotherapy and renal effects in simple hypertension patients and different types of patients

Outcomes	No. of SRs	No. of original studies	No. of cases/controls	Follow-up range (weeks)	Estimate (95%CI)	*P*-value
changes of sCr or eGFR						
aliskiren vs placebo	1	3	6812/5448	NA	0.97 [0.81–1.16]	0.73
BUN > 40 mg / dL						
aliskiren vs placebo	1	NA	1508/753	8	1.50 [0.06–36.75]	0.8
aliskiren vs ARBs	1	NA	4579/1223	8–52	1.16 [0.33–4.06]	0.82
aliskiren vs HCT	1	NA	4579/1113	8–52	0.79 [0.26–2.42]	0.68
Creatinine level > 2.0 mg /dL						
aliskiren vs placebo	1	NA	1508/753	8	1.50 [0.06–36.75]	0.8
aliskiren vs ARBs	1	NA	4579/1223	8–52	0.71 [0.19–2.68]	0.62
aliskiren vs HCT	1	NA	4579/1113	8–52	4.13 [0.24–71.59]	0.33
eGFR < 30 mL /min /1.73 m2						
aliskiren vs ARBs	1	NA	4579/1223	8–52	0.53 [0.13–2.13]	0.37
aliskiren vs HCT	1	NA	4579/1113	8–52	3.16 [0.18–56.09]	0.43
Hyperkalaemia						
aliskiren vs placebo	1	NA	1405/752	8	1.40 [0.51–3.87]	0.52
aliskiren vs ARBs	1	NA	NA	4–8	0.93 [0.51–1.70]	0.82
aliskiren vs HCT	1	NA	5450/1113	NA	0.87 [0.62–1.24]	0.43

**Notes:** Type of metric for comparsions: RR (Risk ratio)

Abbreviations: *NA* Not Accessible, *SA* Systematic review

For patients who are suffered from hypertension and diabetes and/or nephropathy or albuminuria at the same time, our study included two reviews [25, 26].^.^ Results showed that in non-diabetic chronic kidney disease patients, 300 mg aliskiren reduced Urinary Protein Excretion Rate (UPER) better than placebo (*p* < 0.05) and 10 mg perindopril [p = no significance (NS)]. In IgA nephropathy patients, 300 mg aliskiren also reduced Urinary Protein Creatinine Rate (UPCR). In T2DM and hypertension and albuminuria patients, 300 mg aliskiren reduced Urinary Albumin Excretion Rate (UAER) better than placebo (*p* < 0.001), worse than 300 mg irbesartan (*p* = NS). 600 mg aliskiren also performed better than the dose of 300 mg (*p* = NS) and 150 mg (*p* < 0.05) (see Table 7).

**Table 7:** aliskren monotherapy and renal effects for patients who are suffered from hypertension and diabetes and/or nephropathy or albuminuria at the same time

Author	Indication	Total Sample Size	Follow up	Drug Comparison	Primary Outcome - Results
Evangelos C. Rizos, Aris P. Agouridis and Moses S.Elisaf	non-diabetic chronic kidney disease	14	42	placebo vs. aliskiren 300 mg vs. perindopril 10 mg	UPER: −36% for aliskiren and − 25% for perindopril 10 mg Both treatments vs. placebo: *p* < 0.05
	IgA nephropathy	22	36	placebo vs. aliskiren 300 mg	Reduction in UPCR (g/g)
	T2DM and hypertension and albuminuria	26 (crossover design)	4 × 2-months	Placebo vs. aliskiren300mg vs. irbesartan 300 mg	UAER: −48% for aliskiren and − 51% for irbesartan Both treatments vs. placebo: *p* < 0.001
	T2DM and hypertension and albuminuria	26 (crossover design)	4 × 2-months	150 or 300 or 600 mg aliskiren vs. placebo	UAER: −52% for 600 mg, − 48% for 300 mg, − 36% for 150 mg (600 mg vs. 150 mg: *p* < 0.05, 600 mg vs. 300 mg: *p* = NS) All treatments vs. placebo: *p* < 0.001

**Notes:** The comparsions included aliskiren versus placebo, aliskiren versus ARBs and ACEIs.

Abbreviations: *UAER* Urinary Albumin Excretion Ratio, *UPCR* Urinary Protein Creatinine Rate, *UPER* Urinary Protein Excretion Rate

### Prediction intervals and heterogeneity between studies

We calculated 95%PI, the null value was excluded in only 21 meta analyses. Twenty-fivemeta-analyses showed no heterogeneity (I^2^ = 0) for several outcomes. Substantial heterogeneity (50% ≤ I^2^ ≤ 75%) was present in 6 meta analyses addressing clinical outcomes. Considerable heterogeneity (I^2^ > 75%) was present in only three meta-analyses in three outcomes (changes of SBP, DBP when comparing aliskiren150-300 mg with low to high ARBs, BP response rate when comparing aliskiren300mg with placebo).

### Publication bias of included studies

We performed Egger’s regression test in only 36 meta analyses in our reanalysis, because the remaining analyses contained insufficient numbers of studies. In those we analysed, only two had statistical evidence of publication bias (*P* < 0.1). The two are aliskiren150mg versus placebo in BP response rate (*P* = 0.008), aliskiren75mg versus placebo in withdrawal due to adverse events (*P* = 0.008). For those that can be calculated, publication bias of meta analyses included in our study were not existed.

### AMSTAR 2 classification of included studies

When evaluating the important items, most of the studies used a comprehensive literature search strategy (10/14), justified for excluding individual studies (11/14), used a satisfactory technique for evaluating the risk of bias (RoB) in individual studies (9/14), gave thoughts to RoB of original trials when interpreting the results in the review (8/14). All of the included reviews stated explicitly that the review methods were established prior to the conduct and used appropriate methods to combine the results. But few of the studies considered the possibility of publication bias and discussed its likely impact (4/14).

When we examined the study as a whole, however, most of our included reviews had one or more defects in important items. According to the appraisal standard, one review has a critical flaw with or without non-critical weaknesses is considered low, one review has more than one critical flaw with or without non-critical weaknesses is considered critically low [14]. That explains why our included studies are mostly not of high quality. Overall, only three of included studies are rated high, and one is rated moderate, the other ten remaining studies are rated low or critically low (see Table 8 AMSTAR 2 classification of included studies).

**Table 8:** AMSTAR 2 classification of included studies

Study	Year	Item1	Item2	Item3	Item4	Item5	Item6	Item7	Item8	Item9	Item10	Item11	Item12	Item13	Item14	Item15	Item16	Grade
Yongfei Chen [14]	2013	Y	P	N	Y	N	Y	Y	Y	Y	N	Y	Y	Y	Y	N	N	low
Musini VM [8]	2017	Y	Y	Y	Y	Y	Y	Y	Y	Y	Y	Y	Y	Y	Y	Y	Y	high
Gao D [15]	2011	Y	Y	Y	Y	Y	Y	Y	Y	Y	Y	Y	Y	Y	Y	Y	Y	high
Zhenfeng Zheng [16]	2011	Y	Y	N	Y	N	Y	Y	Y	Y	Y	Y	Y	Y	Y	Y	Y	moderate
Verdecchia P [17]	2010	Y	P	N	Y	N	Y	Y	Y	N	Y	Y	N	N	N	N	Y	critically low
Powers B [18]	2011	Y	P	N	Y	N	N	Y	N	Y	Y	Y	Y	Y	Y	N	Y	low
Stanton AV [19]	2010	Y	P	N	N	N	N	P	P	N	Y	Y	N	N	N	N	Y	critically low
Luo Y [20]	2018	Y	Y	Y	Y	N	N	Y	Y	Y	Y	Y	Y	Y	Y	N	Y	low
Zhang JT [21]	2015	Y	Y	Y	Y	Y	Y	Y	Y	Y	N	Y	Y	Y	Y	Y	Y	high
Zheng SL [22]	2017	Y	Y	N	Y	Y	Y	Y	Y	Y	Y	Y	Y	Y	Y	N	Y	low
Gradman AH [23]	2010	Y	Y	N	N	N	N	N	Y	N	Y	Y	N	N	Y	N	N	critically low
White WB [24]	2011	Y	P	N	Y	N	N	N	N	N	Y	Y	N	N	Y	N	Y	critically low
Rizos EC [25]	2012	Y	P	N	N	N	Y	Y	P	N	Y	Y	Y	N	Y	N	Y	critically low
Louvis N [26]	2018	Y	P	N	N	N	Y	N	Y	Y	Y	Y	N	N	Y	N	Y	critically low

**Note:** Abbreviations: *Y* Yes, *N* No, *P* Partial

## Discussion

### Main findings and possible explanations

Aliskiren supplement is commonly considered to be beneficial in the treatment of hypertension, HF and renal dysfunction. Supplement of aliskiren has been the subject of numerous meta-analyses on a varied range of outcomes. We performed this umbrella review to bring the existing evidence together and draw conclusions for the overall effects of AM on clinical outcomes. We identified a large number of meta analyses, systematic reviews and pooled analyses of RCTs with several important distinctive outcomes.

Positive effects of AM in the treatment of hypertension were supported by the findings in our study. Compared with placebo, aliskiren led to a greater degree in lowering BP, a higher rate of BP response and BP control. Compared with ACEIs, It showed a good superiority in BP reduction and produced similar effects as ARBs and HCTZ on three efficacy outcomes.

Aliskiren also showed a good performance in terms of safety and tolerability. The incidence of adverse events, severe adverse events and withdrawal due to adverse events were mostly comparable between aliskiren and placebo or other comparator drugs. The incidence of some specific adverse events, including dizziness, fatigue, nausea, edema, bronchitis, infection, nasopharyngitis and back pain are comparable between aliskiren and placebo, ARBs or HCT [20, 24]. A single meta analysis showed that aliskiren had a larger chance of developing diarrhea than placebo, while only in the high dose group (aliskiren = 600 mg) [8]. However, headache was reported less frequently with aliskiren150mg and 300 mg than with placebo [20]. Hypotension was reported less frequently with AM than with thiazide diuretic monotherapy [24]. Furthermore, a meta analysis including two original studies showed that compared with rampril (a kind of ACEI drug), aliskiren reduced the rate of cough by 67% (RR0.33, 0.22 to 0.49) [19].

The antihypertensive effects of aliskiren may be explained by its association with a decreased generation of Ang I, as it blocks generation of Ang I from angiotensinogen, by inhibiting the active enzymatic site of renin. Furthermore, aliskiren can inhibit renin activity and block RAAS cascade at its primary steps, which provides an appropriate means of RAAS inhibition [7]. However, aliskiren should not be taken at a high dose, aliskiren 600 mg resulted in more frequent and severe adverse events. Aliskiren300mg is the most optimal because it presented significant effects of lowering BP and was safer. In a word, if used properly, aliskiren would be a trustworthy drug for essential hypertension patients.

In HF patients, results showed aliskiren significantly decreased NT-proBNP and PRA levels, increase PRC levels, but had no important influence on aldosterone and BNP levels. NT-proBNP, BNP, PRA, PRC, aldosterone are all biological markers connected tightly with heart failure. Of them, NT- pro BNP and BNP levels are particularly important, and can be used to evaluate the severity of disease [27]. However, when evaluating cardiovascular outcomes in HF patients, our results showed that compared with placebo, aliskiren showed no obvious effects on death rate, the incidence of myocardial infarction and stroke. Compared with enalapril, aliskiren showed no effects on combined CVD mortality and HF hospitalization. The outcomes suggested aliskiren therapy may have good results on some markers, but it would not reduce risk of cardiovascular outcomes, HF hospitalization or total mortality.

Similarly, according to another report, aliskiren led to a significant and sustained reduction in natriuretic peptide levels, but it did not reduce mortality or re-hospitalization rate [28]. The explanation may be that there exists an unknown upper limit for the benefits of RAAS blockade, and over inhibition of it may cause malfunction [29]. Aliskiren, either used alone or in combination with standard medical therapies, is associated with more adverse events, including hyperkalaemia, renal dysfunction and hypotension [30].^.^ These negative events would play a crucial role in offsetting any beneficial effects of aliskiren treatment on HF progression (with or without diabetes), as a result, patients’ major cardiovascular outcomes would not be altered [31].

However, since aliskiren has the possibility to increase the risk of hypotension and hyperkalaemia, it may be expected to apply for HF patients who are suffered from hypertension or hypokalaemia at the same time [32]. Moreover, a small-scale published RCT has initially shown that aliskiren lowered arterial stiffness and left ventricular diastolic function in elderly hypertensive patients during a follow-up of 6 months [33]. The study showed that aliskiren can be indicated for HF with preserved ejection fraction patients. In summary, AM can only be clinically useful for a particular group of HF patients.

In terms of renal effects, for essential hypertension patients, the occurrence of hyperkalemia, blood urea nitrogen (BUN) > 40 mg/dL, sCr > 2.0 mg /dL and eGFR < 30 mL /min /1.73 m^2^ are comparable between aliskiren and placebo, ARBs or HCT. For different types of patients, composite changes of sCr or eGFR are comparable between aliskiren and matched drugs. For patients who are suffered from hypertension and diabetes and/or nephropathy or albuminuria at the same time, our study showed that aliskiren had good performance in changes of surrogate markers. However, as our study included few RCTs and limited participants, examined no hard outcomes such as mortality, major cardiovascular or renal outcomes, the durations of our included trials were short, the results are not convincing.

The little influence of aliskiren effects on renal progression may be explained by its bilateral effects. On the one hand, hypertension can cause glomerular and tubular destruction in the kidneys, accelerating the development of kidney diseases. The decrease of BP has been reported to inhibit the progression of renal failure [34]. Thus, as an effective antihypertensive drug, aliskiren confers some degree of renoprotection. On the other hand, RAAS inhibitors can affect renal hemodynamic mainly through dilation of the efferent arteriole. This can result in reduced intraglomerular pressure [35] and the decline in GFR, the reductions in GFR are presented by an increase in sCr levels. These changes can increase the risk of hyperkalemia and kidney injury occurring, especially in susceptible populations such as CKD (Chronic Kidney Disease), HF and diabetes mellitus [36].

However, our umbrella review identified limited evidence about aliskiren on cardiovascular and renal outcomes, being under-powered to reach certain conclusions. Most reviews available in the literature, comparing aliskiren with other drugs have focused on surrogate outcomes and did not provide robust estimates [37, 38]. Because most chronic CVD or CKD patients are already receiving standard treatment (ACE inhibitors or ARBs). Moreover, the addition of aliskiren would cause great harm to patients, many trails had to be stopped early. ALTITUDE trial [31] and ASTRONAUT trial [36] are two examples, in addition to standard theapy, aliskiren showed similar effects as placebo in cardiovascular and renal outcomes, rate of CVD death and HF hospitalization. In aliskiren group, there was a higher risk of adverse events including stroke, hyperkalemia, hypotension, and renal impairment/renal failure, all-cause mortality was also found to be significantly increased in patients with diabetes but not in those without diabetes. As a consequence, the following ATMOSPHERE trial [39], which compared aliskiren to enalaprial in clinical outcomes in more than 7000 HF patients, was also forced to remove those co-diabetes patients, to minimize possible harm.

Results of our umbrella review are in line with the current guide recommendations. In the latest ACC/AHA guideline [1], DRI aliskiren is classified as one of the effective oral antihypertensive drugs. It is recommended to use once a day, 150-300 mg each time. It is very long acting. After using aliskiren, there is an increased risk of hyperkalemia in CKD or in those on K^+^ supplements or K^+^- sparing drugs. The guideline also pointed out that aliskiren may cause acute renal failure in patients with severe bilateral renal artery stenosis. In the latest ESC/ESH guideline [40], aliskiren was emphasized again that it should be abandoned in pregnancy, it should not be combined with ACEIs or ARBs. In particular, we should be specially noted about contraindications and warnings for aliskiren-containing medicines in patients with diabetes, heart diseases and kidney problems.

### Strengths and limitations

This umbrella review manifests the most comprehensive neutral evaluation of the literature of published associations between AM and related clinical outcomes. When possible, we reanalysed meta-analysis with a comprehensive approach that included the use of random effects analysis, the calculation of predicative intervals and publication bias, to get a better comparison across studies. Another advantage of our study is the stratification of some comparisons by dosage or category of the medication, to make the results more detailed and comprehensive. We also used the latest standard approach (AMSTAR 2) to evaluate quality of included reviews.

However, some unavoidable limitations and caveats are existed in our study. Firstly, in terms of addressing cardiovascular and renal effects, systematic reviews that studied these ranges are number limited or included small participants. Secondly, most of them examined the changes of surrogate markers. No hard outcomes like mortality, major cardiovascular or renal outcomes were examined. Secondly, though we had looked into the original studies of each systematic review, some unpublished data may still be missing, might lead to bias in our overview. Finally, due to the limited number of meta-analyses that studied the association between AM and clinical outcomes, some of included studies were not of high quality, especially the three pooled analyses.

## Conclusion

AM has been investigated for associations with clinical efficacy and safety for the past recent years. We found solid evidence to support the benefits of aliskiren in the treatment of essential hypertension, it can produce significant effects of lowering BP and reliable safety. However, aliskiren presented comparable effects as placebo, other active comparators (ARBs, ACEIs and HCT) in cardiovascular and renal outcomes.

On the one hand, to draw firmer conclusions, we need more RCTs that studied deeply in aliskiren’s efficacy on cardiovascular and renal outcomes, we need studies that can provide real hard outcomes and standardized reporting of analyse. On the other hand, our study is of great clinical value, it will help clinicians and patients weigh up the pros and cons of aliskiren for different diseases, for different types of patients.

## Data Availability

All data used for the umbrella review is contained within the manuscript.

## Abbreviations

AM: Aliskiren monotherapy
HF: Heart failure
BP: Blood pressure
RAAS: Renin-angiotensin-aldosterone system
DRI: Direct renin inhibitor
FDA: Food and Drug Administration
BNP: Brain natriuretic peptide
NT-proBNP: N-terminal prohormone of BNP
PRA: Plasma renin activity
PRC: Plasma renin concentration
ARBS: Angiotensin receptor blockers
ACEIs: Angiotensin-converting enzyme inhibitors
HCT/HCTZ: Hydrochlorothiazide
RCT: Randomized controlled trial
CI: Confidence interval
RR: Relative risk
OR: Odds ratio
WMD: Weighted mean difference
SMD: Standardized mean difference
PI: Predication interval
DBP: Diastolic blood pressure
SBP: Systolic blood pressure
msDBP: Mean sitting DBP
msSBP: Mean sitting DBP
CVD: Cardiovascular disease
sCr: Serum creatinine
eGFR: Estimated glomerular filtration rate
UPER: Urinary protein excretion rate
NS: No significance
UPCR: Reduced urinary protein Creatinine rate
UAER: Urinary albumin excretion rate
RoB: Risk of bias
CKD: Chronic kidney disease
ACC: American college of cardiology
AHA: American Heart Association
ESC: European society of cardiology
ESH: European society of hypertension
